# Chemotherapy Synergizes with Radioimmunotherapy Targeting La Autoantigen in Tumors

**DOI:** 10.1371/journal.pone.0004630

**Published:** 2009-02-27

**Authors:** Fares Al-Ejeh, Jocelyn M. Darby, Michael P. Brown

**Affiliations:** 1 Experimental Therapeutics Laboratory, Hanson Institute, Adelaide, South Australia, Australia; 2 Department of Medical Oncology, Royal Adelaide Hospital and School of Medicine, The University of Adelaide, Adelaide, South Australia, Australia; Genentech, United States of America

## Abstract

**Background:**

To date, inefficient delivery of therapeutic doses of radionuclides to solid tumors limits the clinical utility of radioimmunotherapy. We aim to test the therapeutic utility of Yttrium-90 (^90^Y)-radio-conjugates of a monoclonal antibody, which we showed previously to bind specifically to the abundant intracellular La ribonucleoprotein revealed in dead tumor cells after DNA-damaging treatment.

**Methodology/Principal Findings:**

Immunoconjugates of the DAB4 clone of the La-specific monoclonal antibody, APOMAB®, were prepared using the metal chelator, 1,4,7,10-tetraazacyclododecane-1,4,7,10-tetraacetic acid (DOTA), and then radiolabeled with ^90^Y. Mice bearing established subcutaneous tumors were treated with ^90^Y-DOTA-DAB4 alone or after chemotherapy. Non-radiosensitizing cyclophosphamide/etoposide chemotherapy was used for the syngeneic EL4 lymphoma model. Radiosensitizing cisplatin/gemcitabine chemotherapy was used for the syngeneic Lewis Lung carcinoma (LL2) model, and for the xenograft models of LNCaP prostatic carcinoma and Panc-1 pancreatic carcinoma. We demonstrate the safety, specificity, and efficacy of ^90^Y-DOTA-DAB4-radioimmunotherapy alone or combined with chemotherapy. EL4 lymphoma-bearing mice either were cured at higher doses of radioimmunotherapy alone or lower doses of radioimmunotherapy in synergy with chemotherapy. Radioimmunotherapy alone was less effective in chemo- and radio-resistant carcinoma models. However, radioimmunotherapy synergized with radiosensitizing chemotherapy to retard significantly tumor regrowth and so prolong the survival of mice bearing LL2, LNCaP, or Panc-1 subcutaneous tumor implants.

**Conclusions/Significance:**

We report proof-of-concept data supporting a unique form of radioimmunotherapy, which delivers bystander killing to viable cancer cells after targeting the universal cancer antigen, La, created by DNA-damaging treatment in neighboring dead cancer cells. Subsequently we propose that DAB4-targeted ionizing radiation induces additional cycles of tumor cell death, which further augments DAB4 binding to produce a tumor-lethal ‘genotoxic chain reaction’. Clinically, this approach may be useful as consolidation treatment after a drug-induced cell death among (small-volume) metastatic deposits, the commonest cause of cancer death.

This article is part II of a two-part series providing proof-of-concept for the diagnostic and therapeutic use of the DAB4 clone of the La-specific monoclonal antibody, APOMAB®.

## Introduction

The therapeutic activity of monoclonal antibodies (mAb) may be improved by arming them with additional effector mechanisms [1] such as ionizing radiation that kills neighboring untargeted tumor cells by bystander and/or radiation crossfire effects [2]. The only US Food and Drug Administration (FDA)-approved radioimmunotherapy (RIT) uses anti-CD20 monoclonal antibodies (mAb) armed with ^131^I (tositumomab) or ^90^Y (ibritumomab tiuxetan), which display clinical efficacy even in follicular non-Hodgkin lymphoma (NHL) patients refractory to rituximab [3]. Notwithstanding the clinical utility of CD20-directed RIT for rituximab-refractory NHL, the two approved products have had limited commercial success perhaps because the niche indication for their use necessarily restricts sales, and because the sheer logistical complexity of their application attenuates their clinical acceptance. Moreover, several factors curb the clinical utility of radioimmunotherapy for metastatic carcinoma, which comprises a more populous group of malignancies than lymphoma. Tumor-related factors include radioresistance, and the heterogeneous and low-level expression of target antigens that reduce tumor accumulation of radioimmunoconjugates. Myelosuppression remains the dose-limiting toxicity of radioimmunotherapy [3].

In spite of evasion of apoptosis being recognized as a hallmark of cancer [4], dead cells remain a common feature of many malignancies [5]–[7], and may increase in number after primary chemotherapy [8], [9]. For example, the only approved radioimmunotherapy for carcinoma worldwide is tumor necrosis therapy (TNT). TNT-1 is an ^131^I-labeled chimeric IgG, which was approved by the Chinese State Food and Drug Administration and which produced an overall objective response rate of 34% among patients with advanced lung cancer [2]. Unlike many radioimmunoconjugates that target cell surface antigens, TNT-1 is directed against an intracellular histone/DNA epitope [5], [10], which is present in necrotic and degenerating areas of tumors adjacent to viable tumor cells. Similarly, the 7E11 mAb, which is specific for an internal epitope of the cytoplasmic domain of prostate specific membrane antigen (PSMA^int^), also binds dead cells such as those of the human LNCaP prostate cancer cell line [11]. However, while ^90^Y-labeled 7E11 mAb is ineffective in patients with advanced prostate cancer [12], mAb targeted to the extracellular domains of PSMA display anti-tumor activity [13].

In contradistinction to other nuclear antigens, we discovered that the abundant La ribonucleoprotein (RNP) is overexpressed in malignancy and actively induced in apoptotic malignant cells in response to DNA-damaging treatment [14]. During apoptosis, the La antigen translocates from nucleus to cytoplasm [15], and is fixed in dying cells by transglutaminase 2 (TG2) [14]. As cell membrane integrity is lost during the late phase of apoptosis, cytoplasmic La becomes accessible to binding by specific mAb, which itself in turn becomes crosslinked in the dying cell by TG2 [14]. Together, these characteristics help to explain the preferential and antigen-specific tumor targeting of a La-specific mAb in vivo particularly after cytotoxic chemotherapy [16].

Recently, emerging evidence indicates that impaired clearance of apoptotic cells or defective ‘waste disposal’ contributes to the generation of autoantibodies including those with La/SSB specificity in systemic autoimmune diseases such as systemic lupus erythematosis (SLE) and Sjögren's syndrome [17]. Similarly, the discovery of autoantibodies including La/SSB-specific autoantibodies in sera of cancer patients [18], [19] suggests that autoantibodies also arise as a result of inefficient in vivo clearance of dying cancer cells [20]. Importantly, La-specific antibodies do not appear to have a direct pathogenic role except in the rare condition of congenital heart block, which affects the fetuses of 1–2% of pregnant women who have SLE or Sjögren's syndrome [21].

To develop La-targeted radioimmunotherapy, we optimized conditions for radioimmunoconjugation of the DAB4 clone of the La-specific mAb, which is represented by the APOMAB® trademark [22]. It is important to note that DAB4 originates from a murine autoantibody [23]. The antigen-binding domain of DAB4 has been cloned, sequenced, expressed, and found to bind with low nanomolar affinity to a La epitope, which is highly conserved between humans and rodents (Al-Ejeh et al., unpublished data). Here, using murine lymphoma and carcinoma models, we report therapeutic synergy between La-targeted radioimmunotherapy and DNA-damaging chemotherapy.

## Materials and Methods

### Ethics statement

The Animal Ethics Committee of the Institute of Medical and Veterinary Sciences gave approval for use of the mice. In the use and care of the mice, we followed the humane research principles of replacement, reduction and refinement endorsed by the National Health and Medical Research Council of Australia. Replacement was not possible because there were no alternative techniques to tumor-bearing mice. We achieved a reduction in the numbers of animals used through improved experimental design. Refinement of procedures to improve the welfare of the animals, such as use of analgesics, avoiding significant adverse effects, and enhanced housing conditions, were adopted.

### Preparation of radioimmunoconjugates (RIC)

The La-specific murine mAb (DAB4), the prostate membrane specific antigen (PSMA)-directed mAb (7E11) and their isotype-matched (IgG2a_κ_) control mAb (Sal5) were conjugated to 1,4,7,10-tetraazacyclododedane-*N*,*N*′,*N*″,*N*‴-tetraacetic acid mono-(N-hydroxysuccinimidyl) (DOTA-NHS) ester. DAB4 and Sal5 were radiolabeled with Indium-111 as described [14], [16]. Using identical conditions, DOTA-mAb conjugates were also radiolabeled with Yttrium-90. Radio-doses of ^90^Y-labeled mAb were determined using the AtomLab100 dose calibrator.

### Animal tumor models

The well-characterized EL4 lymphoma model [24] was used as previously modified [16]. Lewis lung carcinomas (LL2) were established in 6–8 week old C57BL/6 (B6) mice by injection of 10^6^ cells in the right flank. Human prostatic (LNCaP) and pancreatic (Panc-1) carcinomas were established in 6–8 week old Balb/c nude mice by injection of 5×10^6^ cells, which were prepared in 50% v/v of matrigel in PBS, in the right flank.

### Dosimetry calculations

C57BL/6 mice with 1 week-old EL4 tumor implants were untreated or given 25 mg/kg cyclophosphamide and 19 mg/kg etoposide (full-dose chemotherapy) by intraperitoneal injection (i.p.i.). ^111^In-DOTA-DAB4 was given by intravenous injection (i.v.i.) in the tail vein to tumor-bearing mice 24 h after chemotherapy, and an organ assay was performed [16]. Briefly, radioactivity in counts per minute (cpm) of harvested organs was divided by the mass to give cpm/g. Then, organ accumulation was expressed as the injected dose per gram (%ID/g) of tissue, which was calculated as the percentage of mass-normalized counts to the total counts (cpm) of ^111^In-DOTA-DAB4 at time 0. Biodistribution studies were done using ^90^Y-DOTA-DAB4 (1.0 MBq). Organ accumulation was calculated as above, however, radioactivity was measured by a TriCarb 3000 beta-counter (PerkinElmer Inc., Wellesley, MA) using a 15–2000 keV counting window. Area-under-curve (AUC) values were calculated from time-activity curves, which were generated from the biodistribution of ^111^In-DOTA-DAB4 or ^90^Y-DOTA-DAB4 over a 96-h analysis period.

### Treatment of tumor-bearing mice

Calipers were used to measure the largest (a) and smallest (b) diameters of the tumors, and tumor volume was calculated according to the equation: *volume = (b^2^×a)/2*. B6 mice bearing EL4 tumors 120–130 mm^3^ in volume were given i.p. 9.5 mg/kg etoposide and 12.5 mg/kg cyclophosphamide (half dose) or 19 mg/kg etoposide and 25 mg/kg cyclophosphamide (full dose). B6 mice bearing LL2 tumors 50–60 mm^3^ in volume were given i.v. 2.5 mg/kg cisplatin and 50 mg/kg gemcitabine on day 1, and 50 mg/kg gemcitabine on day 2. Balb/c nude mice bearing LNCaP and Panc-1 tumors 90–100 mm^3^ and 50–60 mm^3^ in volume, respectively, were given i.v. 1.0 mg/kg cisplatin on days 1 and 7, and 50 mg/kg gemcitabine on days 1, 4, 7, and 10. RIT was given by IVI. The specific activities of ^90^Y–RIC were 65–80 MBq/mg (1.8–2.2 mCi/mg), which were diluted to achieve the specified doses where the final volume was adjusted to 100 µL using PBS. The mean doses (±SEM) administered to the mice weighting 19–21 g were 0.46±0.01, 0.92±0.02, 1.80±0.09, 2.40±0.08 and 3.60±0.1 MBq (*n* = 5). For combination chemotherapy and RIT, RIT was administered 24 h after chemotherapy for the EL4 tumor model, and on day 3 for the LL2 tumor model. For the LNCaP and Panc-1 models, RIT was administered on day 2, and the day 10 dose of gemcitabine (50 mg/kg) was omitted to avoid toxicity otherwise observed (data not shown). Kaplan-Meier survival curves were generated using predefined tumor volumes as endpoints.

### Monitoring of tumor-bearing mice

Mouse weights and tumor volumes were measured every two days in the first two weeks of treatment and then once a week. Mice were euthanized immediately by cervical dislocation or CO_2_ narcosis if a clinical score >3. One point was allocated for each clinical sign: a) weight loss >15% (cf. day 0) that did not reverse within 24 h, b) reluctance to move, eat or drink, c) hunched posture, d) ruffled coat or fur loss, and e) tumor volume >0.5 cm^3^ or tumor size that hindered mobility. If ≥60% of mice in an experimental group had a clinical score >3 then all mice in the group were killed and treatment was denoted as toxic.

An independent veterinary pathologist performed necropsies on EL4 tumor-free mice 61 days after 1.80 and 3.60 MBq ^90^Y-DOTA-DAB4 alone, or 0.46 and 0.92 MBq ^90^Y-DOTA-DAB4 given after chemotherapy (n = 2/group). H&E sections of liver, kidneys, decalcified bone, spleen, small intestine, heart, and lungs were examined. Necropsies were done at the stated times on LL2 tumor-bearing mice from these experimental groups: control (day 6); chemotherapy alone (day 12); ^90^Y-DOTA-DAB4 alone at 0.46, 0.92, 1.80 and 3.60 MBq doses on days 6, 6, 7, and 9, respectively; and chemotherapy combined with ^90^Y-DOTA-DAB4 at the same doses on days 12, 15, 17, and 19, respectively (n>2/group). H&E sections of decalcified bone, lung, heart, small intestine, liver, spleen or kidney were examined.

### Fluorocytometric Analysis of EL4 Lymphoma

Tumors were finely minced with scissors into pieces<10 mm^3^, and a weighed proportion (0.1 g) of tumor mince was suspended in collagenase Type 1 in Hanks' Buffered Salt Solution (HBSS) (10 mL; 2 mg/mL) containing 2.5 mM Ca^2+^. Suspensions were incubated at 37°C with constant rotation for 1 h. Digested tumor cell suspensions were passed sequentially through a series of needles (in order of 19G, 23G, and 25G) to remove coarse materials, and then the last filtrate was centrifuged at 350×*g* for 5 min. Pellets were washed with HBSS (10 mL), resuspended in HBSS (1 mL), and aliquots (100 µL) stained for 30 min. at room temperature (RT) in duplicate with either Sal5 or DAB4 (5 µg/mL). Cells were washed twice with phosphate buffered saline (PBS), and incubated with rabbit anti-mouse IgG Alexa_488_ antibody (2 µg/mL) for 30 min. at RT in the dark. Cells were washed thrice with PBS, resuspended in 7-Amino-Actinomycin D (7-AAD) (2 µg/mL), and analyzed 10 min. later using a FACScan (BD Biosciences, San Jose, CA).

Detergent resistance of residual β-radioactivity in EL4 tumors in vivo was assayed using single cell suspensions in which aliquots were incubated for 10 min. at RT with constant shaking in PBS or a 1% solution of Triton X-100 non-ionic detergent prepared in PBS. Percent triton X-100-resistant radioactivity was calculated as the percentage of ^90^Y-DOTA-DAB4 radioactivity counted in the triton X-100 sample to that in the PBS sample.

### Immunohistochemical detection of biotinylated mAb and caspase-3 activation

In vivo tumor binding of biotinylated Sal5 or DAB4 mAb was detected using immunohistochemistry after Sal5-biotin or DAB4-biotin (100 µg) were given i.v.i. to EL4 tumor-bearing mice, which were untreated or given 25 mg/kg cyclophosphamide and 19 mg/kg etoposide. Biotinylated mAb were given i.v.i. 24 h after chemotherapy and tumors were collected 48 h after biotinylated mAb injection (i.e. 72 h after chemotherapy). Each tumor was bisected, and each half embedded in paraffin and sectioned. Sections were stained with H&E or with 1 µg/mL streptavidin-horse radish peroxidase (HRP). Entire sections were scanned using the DotSlide acquisition program (Soft Imaging System, Olympus, Tokyo, Japan) on a DotSlide BX51 Olympus light microscope (Olympus) at 20× magnification. Scanned sections were visualized using OlyVIA software (Olympus Viewer for Imaging Applications) where images of entire tumors or 10 random regions at 10× or 20× were obtained for analysis using analySIS® software (Soft Imaging System, Olympus). Phase color analysis was performed for all images with pixels defining viable cells as blue-counterstained nuclei and DAB4-biotin or Sal5-biotin bound cells as brown staining from DAB deposition.

For immunohistochemical detection of caspase-3 activation, tumors were collected at the indicated time points, and embedded and sectioned as described above. Sections were stained with H&E or with rabbit IgG raised against activated caspase-3 (1 µg/mL; Chemicon-Millipore, MA) followed by biotin-conjugated anti-rabbit IgG antibody (1 µg/mL; Rockland Inc., PA) then 1 µg/mL streptavidin-HRP. Entire sections were scanned using the DotSlide acquisition program as described above. Phase color analysis was performed for all images using pixels to define the different phases: viable cells as blue-counterstained nuclei, apoptotic cells as brown staining from DAB deposition, and necrotic areas as faint blue areas lacking appropriate nuclear morphology.

### Calculation of tumor doubling time and combination index

The tumor doubling time (TDT) and combination index (CI) were calculated using GraphPad Prism v4.0. TDT values were generated from exponential growth curves, which had been fitted to % change in tumor volume data (*r^2^*>0.70). Our CI calculations were adapted [25] to apply to TDT values. First, the TDT value for untreated mice was subtracted from the TDT value for each treatment group to obtain ‘blanked’ TDT values (TDT_B_). Then, the CI at dose X was calculated as the ratio of TDT_B_ values of combination treatment to individual treatments: CI at radioimmunotherapy dose X = (TDT_B_ combination at radioimmunotherapy dose X)/(TDT_B_ chemotherapy alone +TDT_B_ radioimmunotherapy alone at dose X).

### Statistical analysis

Statistical analyses was performed with GraphPad Prism v4.0 software. Unless otherwise stated, intergroup comparisons were made by two-way analysis of variance (ANOVA), with p<0.05 being considered significant. Kaplan-Meier median survival curves were compared using log-rank (Mantel-Cox) and Gehan-Breslow-Wilcoxon tests, with p<0.05 being considered significant.

## Results

### Specificity, safety, and efficacy of La-targeted delivery of ^90^Y-DOTA-DAB4 to murine EL4 lymphoma grafts in vivo

**Figure 1:**
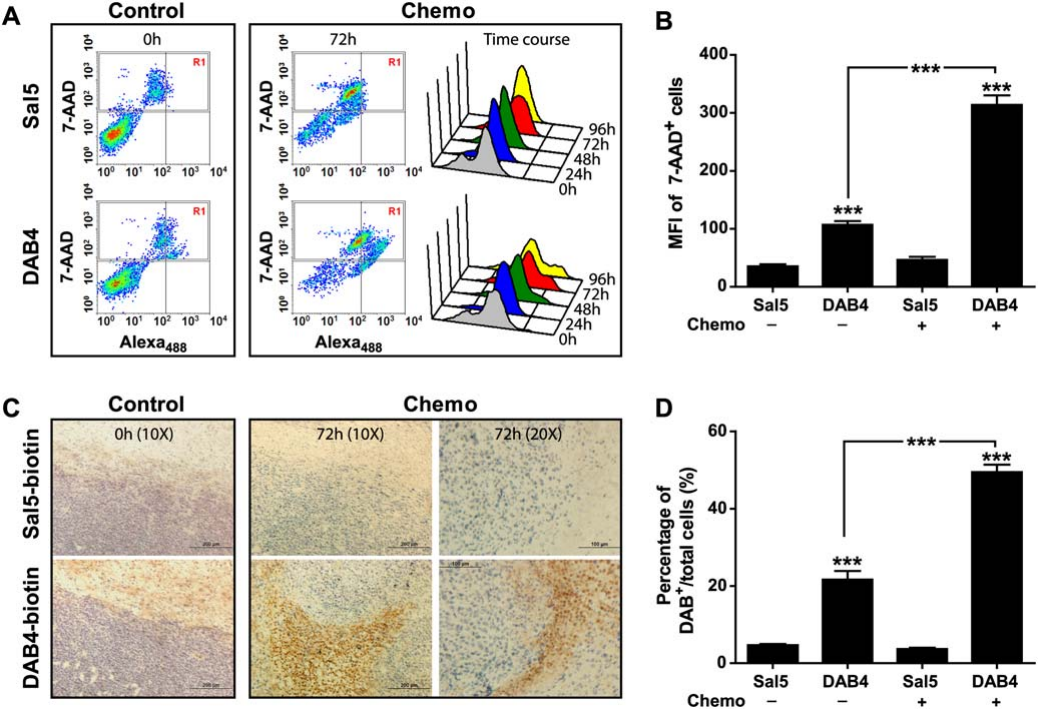
Effects of Chemotherapy on Specific EL4 Tumor Cell Binding of DAB4 Binding Ex Vivo and In Vivo. EL4 tumor-bearing mice (n = 5/group) were killed before (0 h) or 24, 48, 72, and 96 h after treatment with 25 mg/kg cyclophosphamide and 19 mg/kg etoposide i.p.i. A, Single cell suspensions of tumors were stained with 7-AAD and Sal5 or DAB4, and analyzed by flow cytometry. Data shown are representative density plots for Sal5 or DAB4 binding (x-axis) and cell death measured by 7-AAD (y-axis) at 0 h (Control) or 72 h (Chemo). 7-AAD^+^ tumor cells were gated and the mean fluorescence intensities (MFI) of Sal5 or DAB4 staining of these dead cells are displayed in a time course of representative histograms from 0 h (control) up to 96 h after chemotherapy. B, Column graph summarizes MFI±SEM (n = 5/group) for Sal5 and DAB4 staining of 7-AAD^+^ tumor cells at 0 h (control) or at 72 h post-chemotherapy; *** *P*<0.001. C, EL4 tumor bearing mice (n = 3/group) were untreated or treated with cyclophosphamide/etoposide as described above. DAB4-biotin or Sal5-biotin (100 µg) were administered i.v.i. 24 h after chemotherapy and tumors were collected 72 h after chemotherapy (i.e. 48 h after mAb-biotin injection) for immunohistochemical analysis. Representative sections are shown. Scale bar, 200 µm for the four left-hand panels. Scale bar, 100 µm for the two right-hand panels. D, Image and phase color analysis of tumors (n = 3/group) was performed as described in Methods. Data shown are mean percentage (±SEM) of DAB4^+^ cells/total cell number; *** *P*<0.001.

La is a ubiquitous nuclear antigen, which translocates to cytoplasm during apoptosis [26] and which is only accessible to mAb if cells become permeabilized artificially or because of necrosis or late-stage apoptosis [14]. Therefore, it was necessary to establish that uptake of DAB4 by dead tumor cells was specific. First, single EL4 tumor cell suspensions were stained ex vivo with DAB4 or its Sal5 isotype control of irrelevant Salmonella specificity, and analyzed by flow cytometry. As shown in Figure 1A, DAB4 only bound dead tumor cells, and most noticeably after chemotherapy. Moreover, ex vivo tumor cell binding of DAB4 was antigen specific, and augmented by chemotherapy (Figure 1B). Second, we examined the in vivo binding tumor cell of DAB4 or Sal5. EL4 tumor-bearing mice were treated or not with cyclophosphamide/etoposide before intravenous injections of biotinylated forms of DAB4 or Sal5 were given. Biotin was then detected in tumor tissue sections (Figure 1C), and quantification of biotin staining showed that significantly more DAB4-biotin than Sal5-biotin bound EL4 tumors in vivo, particularly in tumors of mice given chemotherapy (Figure 1D). Additional immunofluorescence studies of tumor tissue after in vivo administration of biotinylated mAb indicated that tumor-bound DAB4 was intimately related to cells containing cleaved PARP1, which is recognized as a marker of late apoptosis (Al-Ejeh et al, accompanying manuscript).

These target characteristics together with the more favorable biodistribution of DAB4 conferred by delaying DAB4 administration until 24 hours after chemotherapy (Al-Ejeh et al., accompanying manuscript) suggested that La-directed radioimmunotherapy may have an improved therapeutic ratio. In particular, to understand how best to deliver therapeutic doses of DAB4 mAb labeled with the lethal β-emitting radionuclide, Yttrium-90 (^90^Y), we had studied the biodistribution of radiotracer doses of ^111^In-DOTA-DAB4 in EL4 tumor-bearing mice. These studies indicated that chemotherapy administered 24 hours before the injection of ^111^In-DOTA-DAB4 maximized its tumor uptake and minimized its uptake by critical organs (Al-Ejeh et al., accompanying manuscript).

We evaluated the safety in EL4 tumor-bearing mice of arming La-specific DAB4 mAb or its Sal5 isotype control mAb with doubling doses of ^90^Y. Irrespective of chemotherapy use, mice receiving up to the top 3.60 MBq dose of ^90^Y-DOTA-DAB4 exhibited minimal toxicity, and none of the mice lost >5% of its starting body weight (see Figure S1 online). No late complications were discovered in major organs of tumor-free mice 61 days after treatment.

**Figure 2:**
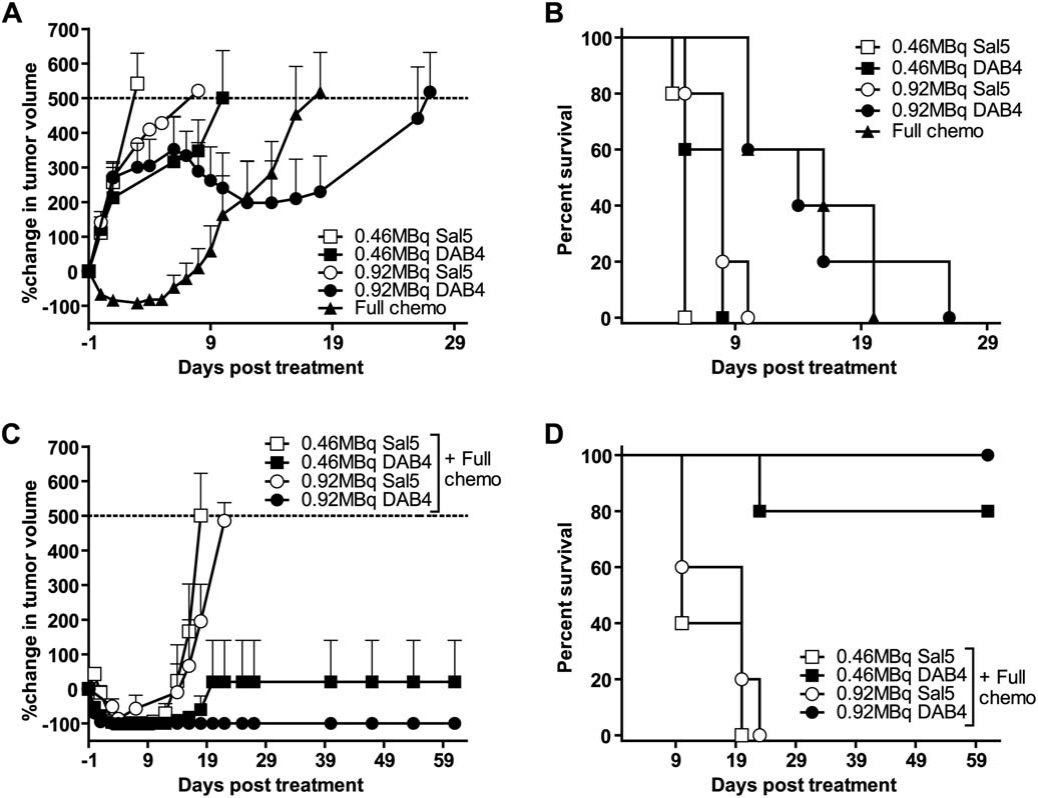
Antitumor Effects in Mice Bearing EL4 Lymphoma, Dose Response, Antigen Specificity, and Chemotherapy Interaction. EL4 tumor-bearing mice were treated with 19 mg/kg etoposide and 25 mg/kg cyclophosphamide i.p.i. (Chemo) or 0.46 MBq and 0.92 MBq doses i.v. of ^90^Y-DOTA-DAB4 or ^90^Y-DOTA-Sal5 alone or 24 h after chemo (n = 5/group). Antitumor effects of Chemo and ^90^Y-radioimmunoconjugates were compared. A, Percentage change in mean tumor volume. B, Kaplan-Meier survival analysis was based on the tumor growth endpoint indicated by dotted line in *A*. Radioimmunotherapy was antigen-specific because median survival times (MST) were extended from 5 d to 8 d for mice treated with 0.46 MBq DAB4 (*P*<0.05 versus 0.46 MBq Sal5), and from 8 d to 14 d for mice treated with 0.92 MBq DAB4 (*P*<0.01 versus 0. 92 MBq Sal5). Antitumor effects of ^90^Y-radioimmunoconjugates given 24 h after Chemo were compared. C, Percentage change in mean tumor volume. D, Kaplan-Meier survival analysis was based on the tumor growth endpoint indicated by dotted line in C. Antigen-specific effects of radioimmunotherapy on survival were highly significant for 0.46 MBq DAB4 and 0.92 MBq ^90^Y-DOTA-DAB4 (*P*<0.001 versus 0.46 and 0.92 MBq ^90^Y-DOTA-Sal5), by 61 d post-chemotherapy. Error bars for all graphs; ±SEM.

Antigen-specific targeting of ^90^Y-radioimmunotherapy was demonstrated irrespective of chemotherapy use (Figure 2). Although neither 0.46 nor 0.92 MBq ^90^Y-DOTA-Sal5 altered the EL4 tumor growth rate, treatment of mice with 0.92 MBq of ^90^Y-DOTA-DAB4 significantly retarded tumor growth and increased median survival time (MST) from 8 to 14 days (Figure 2A&B). At higher (1.80 or 3.60 MBq) doses of ^90^Y-DOTA-DAB4, all mice (n = 5/group) remained tumor-free 61 days after treatment (data not shown). Moreover, giving the lower (0.46 and 0.92 MBq) doses of ^90^Y-DOTA-DAB4 24 hours after chemotherapy rendered 80% and 100% of mice, respectively, tumor-free 61 days after treatment (Figure 2C&D), indicating that chemotherapy effectively reduced the therapeutic dose of ^90^Y-DOTA-DAB4 by four-fold (1.80 MBq alone cf. 0.46 MBq with chemotherapy).

### Associations of effective treatment in EL4 lymphoma-bearing mice, ^90^Y dosimetry, tumor cell death, induction of La target, and tumor retention of DAB4

Although dosimetry of ^111^In-DOTA-DAB4 in EL4 tumor-bearing mice may provide a useful surrogate of ^90^Y dosimetry [27], we were surprised to find that, independently of chemotherapy use, the actual tumor accumulation of ^90^Y was greater than that estimated from dosimetry of ^111^In-DOTA-DAB4 (Table 1). Therefore, we inferred that La-targeting of ^90^Y-DOTA-DAB4 begat its own tumor uptake.

**Table 1:** Comparison of Treatment Effects on the Relative Biodistribution of ^111^In-DOTA-DAB4 and ^90^Y-DOTA-DAB4.

Cumulative organ uptake of DAB4 - mean %ID.g/h (±SEM) for:	Treatment group	*tumor	blood	liver	§spleen	kidney
**γ-counts from ^111^In-label**	**Untreated**	1370 (±60)	1900 (±60)	760 (±40)	680 (±20)	670 (±40)
	**Chemo**	2454 (±30)	1820 (±100)	720 (±100)	730 (±30)	600 (±90)
**β-scintillation counts from ^90^Y-label**	**RIT**	1920 (±120)	1910 (±40)	840 (±40)	950 (±40)	830 (±60)
	**ChemoRIT**	3400 (±200)	1930 (±100)	850 (±40)	960 (±40)	830 (±30)

EL4 tumor-bearing mice were untreated, or treated with 19 mg/kg etoposide and 25 mg/kg cyclophosphamide chemotherapy i.p.i. Separate experiments were conducted under similar conditions: 1.0 MBq ^111^In-DOTA-DAB4 was given i.v.i. to untreated mice or mice 24 h after chemotherapy (Chemo), and 1.0 MBq ^90^Y-DOTA-DAB4 was given i.v.i. to mice as radioimmunotherapy (RIT) or to mice 24 h after chemotherapy as ChemoRIT. Organ assays were performed 3, 24, 48, 72, and 96 h after radioimmunoconjugate injection (n = 5 per time point per treatment group). As a measure of cumulative DAB4 uptake, time-activity curves (see Figure S2 online for detailed organ-specific data) for tumor, blood, liver, spleen, and kidney were used to calculate the mean area under the curve (AUC) for the percentage of injected dose per gram of tissue per hour (%ID.g/h) (as described in Methods).

* In mice not given Chemo, tumor accumulation of the high-energy β-emitting ^90^Y significantly exceeded that of the comparable dose of the low-energy γ-emitting ^111^In (*P*<0.001). In mice given Chemo, tumor accumulation of ^90^Y was significantly higher either than that of ^111^In or of ^90^Y given as RIT alone (*P*<0.001).

§ Significantly more ^90^Y-DOTA-DAB4 accumulated in spleens of mice given RIT than in spleens of control mice given a comparable dose of ^111^In-DOTA-DAB4 (*P*<0.05).

**Figure 3:**
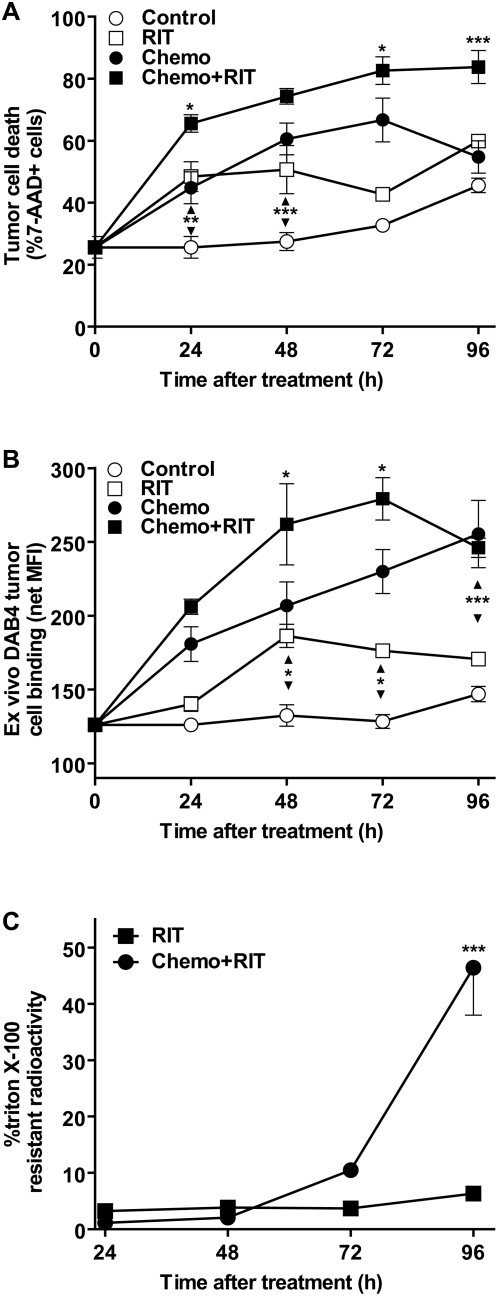
Comparisons of Ex Vivo Analyses of Tumor Cell Death, DAB4 Binding, and Radioactivity in Mice Bearing EL4 Lymphoma. EL4 tumor-bearing mice were untreated (Control), or treated with 19 mg/kg etoposide and 25 mg/kg cyclophosphamide i.p.i. alone (Chemo), 0.92 MBq ^90^Y-DOTA-DAB4 given i.v.i. alone (RIT) or 24 h after chemotherapy (Chemo+RIT). Control and Chemo mice were given 1.0 MBq ^111^In-DOTA-DAB4 i.v.i. (24 h after Chemo). A, Comparison of treatment effects on ex vivo FACS analysis of tumor cell death. Mean percentage 7-AAD^+^ dead EL4 tumor cells. As indicated, radioimmunotherapy produced significantly more tumor cell death at early time points (**P<0.01, *** *P*<0.001 versus Control), and Chemo+RIT induced greater tumor cell death overall (* *P*<0.05 and *** *P*<0.001 versus all other treatments). Note that for Chemo+RIT group, Chemo was given at −24 hour time point on abscissa. B, Comparison of treatment effects on ex vivo FACS analysis of DAB4 tumor cell binding. Ex vivo DAB4 binding to 7-AAD^+^ dead cells was measured as the net mean fluorescence intensity (MFI) after subtraction of MFI values for Sal5 binding (mean of duplicate stains from 3 mice). As indicated, RIT itself augmented per cell binding of DAB4 (**P<0.05 versus Control), whereas Chemo+RIT promoted greater DAB4 binding overall (*P<0.05 versus all other treatments). Note that for Chemo+RIT group, Chemo was given at −24 hour time point on abscissa. *C*, Effect of ^90^Y-DOTA-DAB4 on EL4 tumor cell retention of radioactivity after treatment with the non-ionic detergent, triton X-100. Mean percentage of detergent-resistant radioactivity was calculated from the ratio of radioactivity after triton X-100 treatment to total tumor radioactivity. Chemo+RIT produced significant retention of tumor radioactivity (*P*<0.001 versus RIT alone). Error bars for all graphs; ±SEM.

To investigate factors underlying this phenomenon, we analyzed the frequency of tumor cell death. In the 24–48 hours post-treatment, all therapies yielded a significantly higher frequency of 7-AAD^+^ EL4 tumor cells than observed in control mice (Figure 3A). Strikingly, curative treatment using ^90^Y-DOTA-DAB4 24 hours after chemotherapy produced a cumulative increase in tumor cell death whereas neither sub-curative treatment using chemotherapy or 0.92 MBq ^90^Y-DOTA-DAB4 produced a sustained level of tumor cell death (Figure 3A). Although analysis of ex vivo binding of DAB4 to dead EL4 tumor cells confirmed that DNA-damaging chemotherapy induced the La target [16], giving ^90^Y-DOTA-DAB4 24 hours after chemotherapy augmented ex vivo DAB4 binding significantly more than either treatment alone (*P*<0.05) (Figure 3B). Ex vivo tumor binding of DAB4 was compared with that of TNT1 mAb. Both mAb bound 7-AAD^+^ EL4 cells but only the mean fluorescence intensity (MFI) of DAB4 binding significantly increased with time post-chemotherapy whereas TNT1 MFI declined significantly over time (data not shown).

Since our previous in vitro data indicated that DAB4 became crosslinked in apoptotic tumor cells [14], we determined if intratumoral radioactivity was detergent-resistant. After combination treatment, although significantly more EL4 tumor radioactivity accumulated than with ^90^Y-DOTA-DAB4 alone (data not shown), significantly more detergent-resistant radioactivity was found 96 hours after combination treatment (*P*<0.001) (Figure 3C). Since significantly higher splenic accumulation of ^90^Y-DOTA-DAB4 was detected 48 hours after its injection than would have been expected from the dosimetry of ^111^In-DOTA-DAB4 in control mice (Table 1), splenic accumulation of ^90^Y-DOTA-DAB4 may represent progressive trapping of dead radiolabeled cells by the red pulp of the spleen.

### Safety and efficacy of La-targeted delivery of ^90^Y-DOTA-DAB4 to syngeneic murine lung carcinoma and human prostatic and pancreatic cancer cell xenografts in vivo

**Figure 4:**
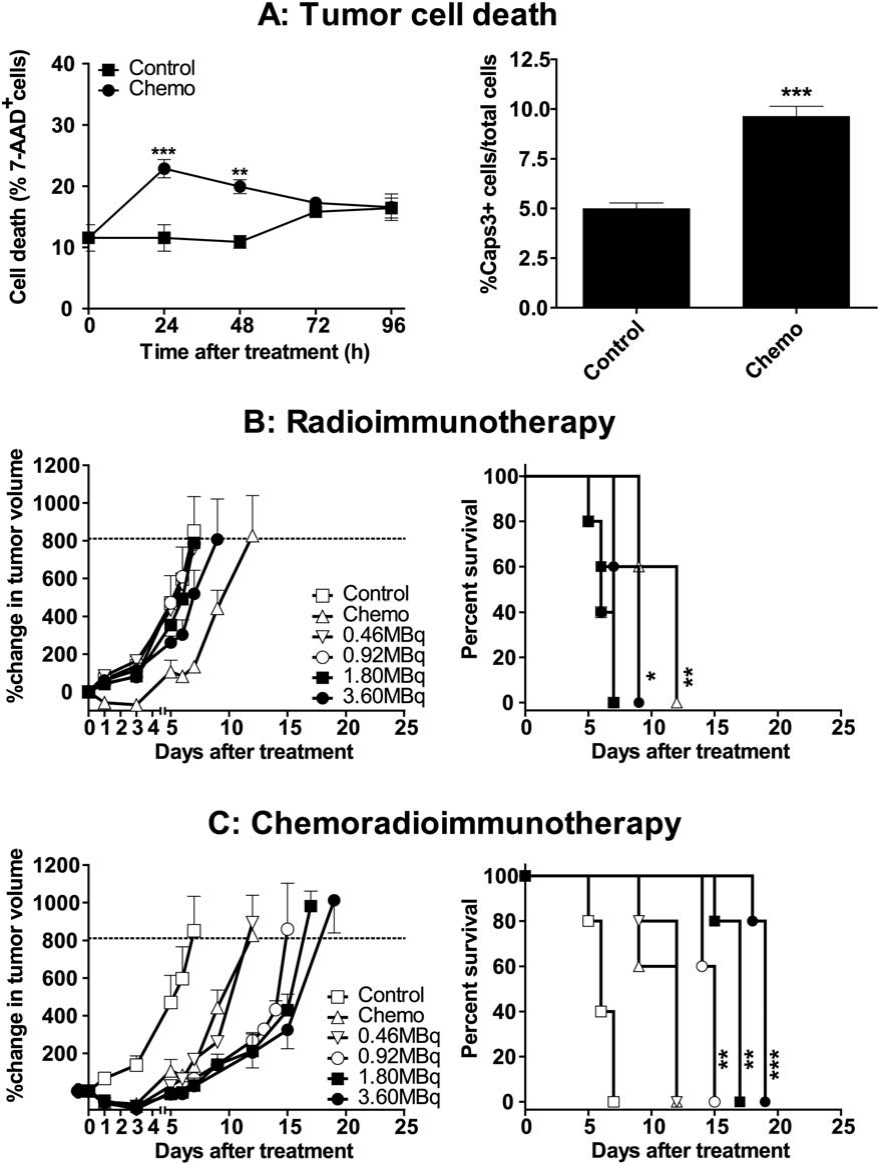
Antitumor Effects in Mice Bearing LL2 Carcinoma, Tumor Cell Death, and Dose Response. A, Analysis of LL2 tumor cell death. LL2 tumor-bearing mice were untreated (Control), or treated i.v.i. with 2.5 mg/kg cisplatin and 50 mg/kg gemcitabine on day 1, and 50 mg/kg gemcitabine on day 2 (Chemo). Left-hand panel, mean percentage 7-AAD^+^ cells in LL2 single tumor cell suspensions (n = 3/group). As indicated, chemotherapy induced significantly more tumor cell death (** *P*<0.01, *** *P*<0.001 versus Control). Right-hand panel, percentage of mean number of activated caspase-3^+^ cells divided by total cell number in LL2 tumor sections from Control mice and mice 48 h after Chemo (n = 5/group). Chemotherapy significantly augmented caspase-3 activation (*P*<0.001 versus Control; two-tailed student's *t*-test). B, Comparison of antitumor effects of chemotherapy and ^90^Y-DOTA-DAB4. LL2 tumor-bearing mice were untreated (Control), treated i.v.i. either with Chemo as in *A* or 0.46 MBq, 0.92 MBq, 1.80 MBq, or 3.60 MBq ^90^Y-DOTA-DAB4 (n = 5/group). Left-hand panel, mean percentage change in tumor volume. Right-hand panel, Kaplan-Meier survival analysis based on the tumor growth endpoint indicated by dotted line in left-hand panel. As indicated, only Chemo and 3.60 MBq ^90^Y-DOTA-DAB4 extended survival (*P<0.05, ** *P*<0.01 versus Control). C, Comparison of antitumor effects of Chemo alone or combined with ^90^Y-DOTA-DAB4. LL2 tumor-bearing mice were untreated (Control), treated i.v.i. either with Chemo alone as in *A* or 0.46 MBq, 0.92 MBq, 1.80 MBq, or 3.60 MBq ^90^Y-DOTA-DAB4 given 24 h after Chemo (n = 5/group). Left-hand panel, mean percentage change in tumor volume. Right-hand panel, Kaplan-Meier survival analysis based on the tumor growth endpoint indicated by dotted line in left-hand panel. As indicated, 0.92 MBq, 1.80 MBq, and 3.60 MBq doses of ^90^Y-DOTA-DAB4 given 24 h after Chemo prolonged survival (**P<0.01, *** *P*<0.001 versus Chemo). Error bars for all graphs; ±SEM.

Next, we turned to relatively radio- and chemo-resistant murine Lewis lung (LL2), and human prostatic (LNCaP) and pancreatic (Panc-1) carcinoma models. Chemotherapy produced a small albeit significant increase in LL2 tumor cell death of 10–15%, which corresponded to an 8–10% increase in the ratio of activated caspase-3^+^ cells to total cells (Figure 4A). First, we determined that the maximum tolerated dose (at which no toxicity-related deaths occurred) was 5.0 MBq for ^90^Y-DOTA-DAB4 used alone or 24 hours after chemotherapy. Subsequently, we analyzed the safety and efficacy of ^90^Y-DOTA-DAB4 alone or with chemotherapy. Importantly, no acute pathology was observed in the major organs of treated LL2 tumor-bearing mice at each of the indicated time points (dotted line, Figure 4B and C).

Although chemotherapy significantly extended the median survival time (MST) of LL2 tumor-bearing mice to 12 days (*P*<0.01 versus control, 6 days), only the 3.60 MBq dose of ^90^Y-DOTA-DAB4 extended MST to 9 days (*P*<0.05 versus control; Figure 4B). Antitumor activity of DAB4 depended on ^90^Y labeling because 50 µg DAB4-DOTA (equivalent to the mAb mass in 3.60 MBq ^90^Y-DOTA-DAB4) did not delay tumor regrowth whether it was used alone or 24 hours after chemotherapy. In contrast and notwithstanding the relatively low levels of chemotherapy-induced tumor cell death, 0.92, 1.80, and 3.60 MBq ^90^Y-DOTA-DAB4 24 hours after chemotherapy extended MST to 15, 17, and 19 days, respectively (*P*<0.01, *P*<0.01, *P*<0.001, respectively, versus control; Figure 4C).

**Figure 5:**
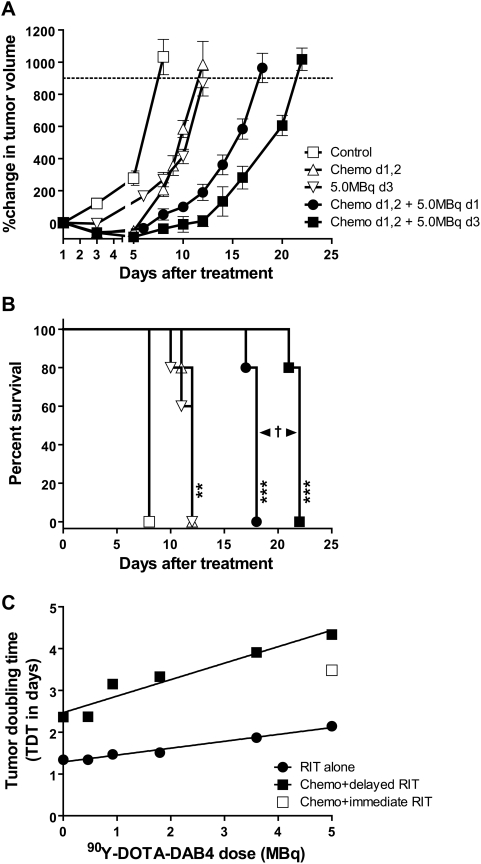
Antitumor Effects in Mice Bearing LL2 Carcinoma, ^90^Y-DOTA-DAB4 Schedule, and Chemotherapy Interaction. LL2 tumor-bearing mice were untreated (Control), treated i.v.i. with 2.5 mg/kg cisplatin and 50 mg/kg gemcitabine on day 1, and 50 mg/kg gemcitabine on day 2 (Chemo d1, 2), or 5.0 MBq of ^90^Y-DOTA-DAB4 i.v.i. alone on day 3 (5.0 MBq d3) or i.v.i. immediately after Chemo (Chemo d1, 2+5.0 MBq d1) or 24 h after Chemo on day 3 (Chemo d1, 2+5.0 MBq d3) (n = 5/group). A, Mean percentage change in tumor volume. B, Kaplan-Meier survival analysis based on the tumor growth endpoint indicated by dotted line in *A*. As indicated, all treatments prolonged survival (**P<0.01, *** *P*<0.001 versus Control), but 5.0 MBq ^90^Y-DOTA-DAB4 was more effective when given 24 h rather than immediately after Chemo (^†^ p<0.01). C, Effects of treatments on doubling times of LL2 tumors. Mean LL2 Tumor Doubling Time (TDT) were derived for treatments outlined in *A* and *B* and plotted as a function of ^90^Y-DOTA-DAB4 dose for (•) radioimmunotherapy (RIT) alone, (□) RIT immediately after Chemo, or (▪) RIT 24 after Chemo. Note that Control and Chemo only mice received 0 MBq RIT. Error bars for all graphs; ±SEM.

Since administration of DAB4 24 hours after chemotherapy enhanced both tumor uptake and clearance from blood and normal organs (Al-Ejeh et al., accompanying manuscript), we investigated the therapeutic effects of varying the schedule of administration (Figure 5). The MST of LL2 tumor-bearing mice was 22 days when 5.0 MBq ^90^Y-DOTA-DAB4 was given 24 hours after chemotherapy compared with 18 days when the dose was given immediately after chemotherapy (*P*<0.01; Figure 4B). As illustrated in Figure 5C, LL2 growth data may also be represented in the form of a tumor doubling time (TDT), which was a linear function of ^90^Y-DOTA-DAB4 dose. While chemotherapy itself further increased TDT, chemotherapy added to radioimmunotherapy augmented the linear dependence of TDT on ^90^Y-DOTA-DAB4 dose by 3.0 (±0.3)-fold (Figure 5C). Using TDT values, we calculated a combination index for chemotherapy and radioimmunotherapy to show that combination treatment using >0.46 MBq ^90^Y-DOTA-DAB4 was supra-additive or synergistic (see Table S1 online).

**Figure 6:**
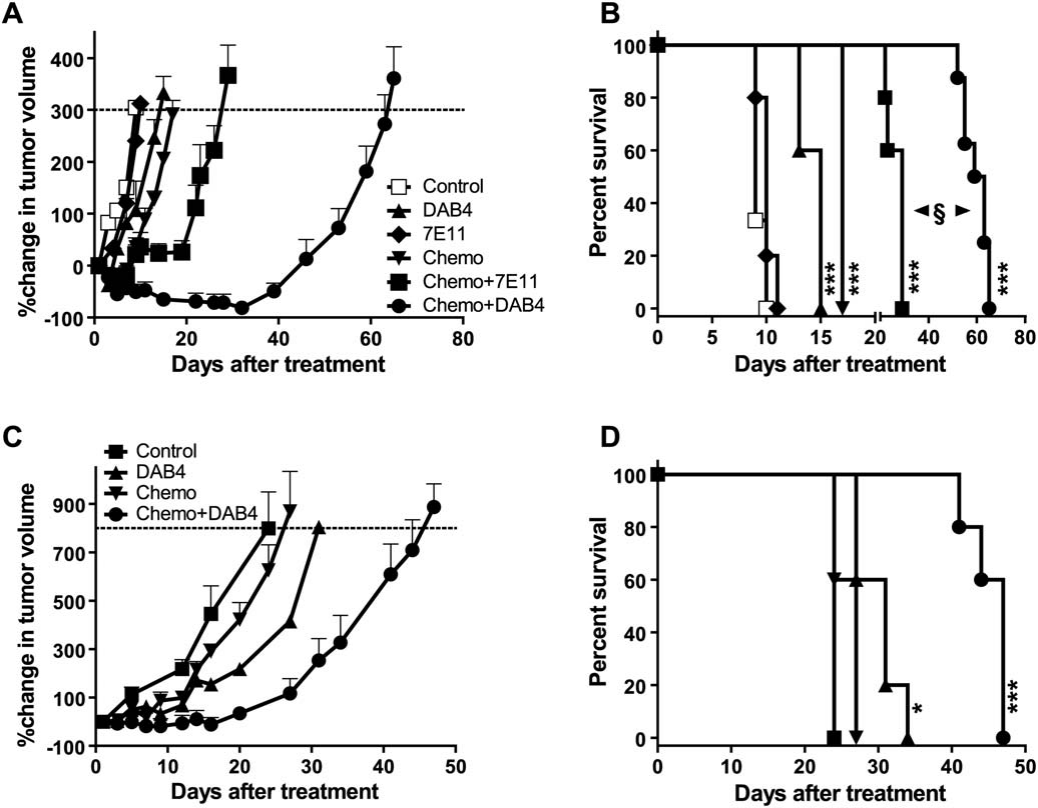
Antitumor Effects in Mice Bearing Human Prostatic or Pancreatic Cell Line Xenografts, Dose Response, and Chemotherapy Interaction. Balb/c nude mice with established subcutaneous LNCaP or Panc-1 tumors were untreated (Control), treated i.v.i. with 1.0 mg/kg cisplatin on days 1 and 7, and 50 mg/kg gemcitabine on days 1, 4, 7, and 10 (Chemo), 2.40 MBq of ^90^Y-DOTA-DAB4 i.v. alone (DAB4), or 24 h after Chemo (Chemo+DAB4). LNCaP-tumor bearing mice were treated i.v. with 2.40 MBq of ^90^Y-DOTA-7E11 alone (7E11), or 24 h after Chemo (Chemo+7E11) (n = 5/group). Note that in chemo+RIT groups, day 10 gemcitabine dose was omitted because of previously observed toxicity. Antitumor effects of ^90^Y-radioimmunoconjugates on LNCaP xenograft growth were compared. A, Mean percentage change in tumor volume. B, Kaplan-Meier survival analysis was based on tumor growth endpoint indicated by dotted line in *A*. As indicated, all treatments except 2.40 MBq ^90^Y-DOTA-7E11 prolonged survival (*** *P*<0.001 versus Control) whereas 2.40 MBq ^90^Y-DOTA-DAB4 24 h after Chemo prolonged the survival of mice more than any other treatment including 2.40 MBq ^90^Y-DOTA-7E11 24 h after Chemo (^§^ *P*<0.01). Antitumor effects of chemotherapy and ^90^Y-DOTA-DAB4 on Panc-1 xenograft growth were compared. C, Mean percentage change in tumor volume. D, Kaplan-Meier survival analysis was based on the tumor growth endpoint indicated by dotted line in *C*. As indicated, ^90^Y-DOTA-DAB4 alone or after Chemo prolonged survival (*P<0.05, *** *P*<0.001 versus Control). Error bars for all graphs; ±SEM.

Finally, we analyzed the interaction between radiosensitizing cisplatin/gemcitabine chemotherapy and a 2.40 MBq dose of ^90^Y-DOTA-DAB4 in two human carcinoma xenograft models. Like LL2 carcinoma, chemotherapy doubled the frequency of tumor cell death from 5.3±0.5% to 10.8±0.8%, and from 8.2±0.5% to 18±2%, in xenografts of LNCaP and Panc-1 cancer cell lines, respectively, 72 hours post-treatment. Despite the low-level chemotherapy-induced tumor cell death in LNCaP tumor-bearing mice, 2.40 MBq ^90^Y-DOTA-DAB4 and chemotherapy increased MST to 15 and 17 days, respectively (*P*<0.001 versus control, 9 days; Figure 6B). In contrast, 2.40 MBq ^90^Y-DOTA-7E11, which is directed against the internal epitope of PSMA expressed by LNCaP cells, did not extend the MST (10 days) of LNCaP tumor-bearing mice (Figure 6B). Furthermore, 2.40 MBq ^90^Y-DOTA-DAB4 given 24 hours after chemotherapy produced a highly significant extension of MST to 61 days (*P*<0.0001 versus control, and *P*<0.001 versus any other treatment including 2.40 MBq ^90^Y-DOTA-7E11 given 24 hours after chemotherapy; Figure 6B).

In the Panc-1 model, although chemotherapy did not influence survival, 2.40 MBq ^90^Y-DOTA-DAB4 extended MST to 31 days (*P*<0.001 versus control, 24 days; Figure 6D), and 2.40 MBq ^90^Y-DOTA-DAB4 24 hours after chemotherapy produced significant extension of MST to 47 days (*P*<0.001 versus control, and *P*<0.01 versus any other treatment; Figure 6D).

Combination index calculations indicated a distinctly supra-additive interaction between chemotherapy and DAB4-mediated radioimmunotherapy in both xenograft models whereas chemotherapy synergized modestly with radioimmunotherapy targeted by PSMA^int^-specific 7E11 mAb in the LNCaP model (see Table S1 online).

## Discussion

We have developed a safe and effective means of delivering systemic cytotoxic chemotherapy and internal ionizing radiation. The safety of the technology was evident by its lack of clinically significant acute and late toxicities and its efficacy by clinically meaningful antitumor activity in mice bearing four different grafts of lymphoma or carcinoma. The therapeutic effect of La-directed radioimmunotherapy was antigen-specific and most pronounced when given after chemotherapy.

In the chemo-responsive [24] and radio-responsive [28] EL4 lymphoma model, we showed that lymphoma-eradicating therapy depended on either the higher 1.80 and 3.60 MBq doses of ^90^Y-DOTA-DAB4 alone or the lower 0.46 and 0.92 MBq doses of ^90^Y-DOTA-DAB4 given 24 hours after chemotherapy. This curative chemoradioimmunotherapy was associated with sustained high levels of both tumor cell death and ex vivo binding dead tumor cell binding of DAB4. Conversely, although the components of the combined therapy individually produced similar extensions of survival of tumor-bearing mice, neither radioimmunotherapy with 0.92 MBq ^90^Y-DOTA-DAB4 nor chemotherapy successfully eradicated lymphoma. In comparison with curative combined therapy, subcurative lymphoma therapy using either 0.92 MBq ^90^Y-DOTA-DAB4 or chemotherapy alone induced lower levels of tumor cell death. Therefore, individual treatment at these doses may have failed to cure mice because the stabilization or reduction in tumor cell death at 72–96 hours post-treatment reflected tumor cell repopulation as well as dissipating cytotoxic activity.

Consequently, we hypothesize that curative lymphoma therapy depended on a first step of “target creation” that then produced a second and self-amplifying step of ^90^Y-DOTA-DAB4 binding, which we have styled as a ‘genotoxic chain reaction’. Our data suggest that the number of new DAB4 binding targets created by chemotherapy or radioimmunotherapy derived from an increased number of dead tumor cells harboring La antigen targets together with increased availability of La antigen targets in each dead tumor cell. For example, in the case of curative single 1.80 or 3.60 MBq doses of ^90^Y-DOTA-DAB4, the number of La antigen targets present in dead cells of untreated EL4 tumors enabled initial binding of ^90^Y-DOTA-DAB4. Subsequently, the radiation crossfire of the high-energy β-emissions was sufficient to kill surrounding viable EL4 lymphoma cells, and in turn generated further rounds of ^90^Y-DOTA-DAB4 recruitment to the tumor.

Additional support for this hypothesis is drawn from the observation that EL4 tumors accumulated significantly greater amounts of ^90^Y-DOTA-DAB4 than would have been predicted by the dosimetry of ^111^In-DOTA-DAB4. Moreover, the finding of detergent-resistant retention of DAB4-targeted radioactivity in EL4 tumors only when ^90^Y-DOTA-DAB4 was given after chemotherapy rather than by itself suggests that tumor accretion of DAB4-targeted radioactivity resulted from increased transglutaminase 2 activity [14]. We propose that this phenomenon may be specific to malignant tissue because we did not find any detergent-resistant accumulation of ^90^Y-DOTA-DAB4 in normal tissues after chemotherapy. In particular, although ^90^Y-DOTA-DAB4 accumulated significantly more in spleen than expected from the dosimetry of ^111^In-DOTA-DAB4, none of this accumulation was detergent-resistant. Finally, recent data from studies of the immunopathogenesis of congenital heart block in which La-specific antibodies were shown to inhibit phagocytosis of fetal cardiomyocytes in vitro [29] suggests that DAB4-mediated inhibition of the in vivo clearance of DAB4-bound dead tumor cells may also prolong tumor retention of ^90^Y-DOTA-DAB4.

In stark contrast to the 70% frequency of chemotherapy-induced EL4 lymphoma cell death, we found that cisplatin and gemcitabine chemotherapy approximately doubled tumor cell death rates among LL2 tumors and human tumor xenografts so that these rates remained low at clinically relevant levels of ≤20% [30]. Nonetheless, we presume that endogenous levels of tumor cell death were sufficient to enable binding of ^90^Y-DOTA-DAB4, which was effective as monotherapy in all carcinoma models. In addition, administering ^90^Y-DOTA-DAB4 after chemotherapy produced greater than additive effects on tumor growth delay and survival in all carcinoma models, which we hypothesize resulted at least in part from increased levels of chemotherapy-induced tumor cell death. Although we have not formally investigated this possibility, radiosensitizing cisplatin and gemcitabine chemotherapy may effectively lower the threshold for radiation-induced carcinoma cell death as suggested for non-cytotoxic doses of gemcitabine [31]. Hence, future studies will aim to correlate antitumor effects of combination treatment with measures of tumor cell death, ex vivo tumor cell binding of DAB4, and tumor accumulation of DAB4.

We found that combination treatment produced greater increments in LL2 tumor doubling time than ^90^Y-DOTA-DAB4 alone. However, despite doubling doses of ^90^Y-DOTA-DAB4, the rate of increase in TDT after combination treatment was less than might be expected. Perhaps, as chemotherapy shrinks the tumor, the maximum 12 mm tissue penetration depth of ^90^Y-derived β-particles results in much of the energy emanating from DAB4-bound dead tumor cells depositing beyond the confines of the shrinking tumor. Conversely, the maximum 2.5 mm tissue penetration depth and the longer physical half-life of Lutetium-177-derived β-particles suggest that the efficacy of radioimmunotherapy dose escalation may be improved if the deposition of energy within the shrinking LL2 tumor mass is more efficient [32]. Having established preclinical proof-of-concept for La-directed β-emitting radioimmunotherapy, as a logical extension, we hypothesize that high-energy α-particles originating from a dead-cell source of a La-bound α-emitting radionuclide will destroy nearby cancer stem cells in small-volume (≤1 mm^3^) cohesive masses of metastatic carcinoma [33], [34].

Similar to the mechanism proposed for therapeutic synergy in the EL4 lymphoma model, we propose that the radiosensitizing chemotherapy facilitated a dose-dependent and self-reinforcing accretion of ^90^Y-DOTA-DAB4 in the carcinoma models. In support of this concept, we found that, irrespective of chemotherapy use, matched radiation doses of ^90^Y-DOTA-7E11, which also targets dead PSMA^int+^ LNCaP cells, produced appreciably less inhibition of LNCaP tumor regrowth than ^90^Y-DOTA-DAB4. Therefore, we propose that *only* binding of dead LNCaP cells by ^90^Y-DOTA-DAB4 created more targets for its own binding because antibody-directed β-radiation induced and revealed significantly more La antigen targets than PSMA^int^ targets in the dead LNCaP cells. Interestingly, expression of PSMA may be lost in LNCaP tumors borne by nude mice after therapeutic intervention (K, Chester, personal communication, University College London Cancer Institute, UK).

Although the inherently greater chemo- and radio-sensitivity of EL4 lymphoma, which is reflected in its brisk and presumably P53-mediated apoptosis [35], may help to explain EL4 tumor eradication by the combination of chemotherapy and low doses of La-directed radioimmunotherapy, other factors may account for the reduced and differential sensitivity to this combination treatment of the carcinoma cell lines, LL2, in particular. First, since the LL2 model is syngeneic, its biological aggressiveness compared with the LNCaP and Panc-1 xenograft models may result from the greater compatibility between tumour and host tissues in a syngeneic model. Second, P53 is mutant in the LL2 [36] and Panc-1 [37] tumors whereas P53 is wild type in the more treatment responsive LNCaP tumor model [38]. Tumor doubling time is a third factor to be considered in conjunction with the other factors, particularly because the LL2 tumor has the shortest doubling time. The doubling times of untreated LL2, LNCaP, and Panc-1 tumors were 1.34±0.02, 1.81±0.01, and 4.44±0.02 days, respectively (see Table S1 online).

Finally, we believe that the schedule of chemo-radioimmunotherapy is critically important for both its safety and efficacy. Our companion studies of the biodistribution of ^111^In-DOTA-DAB4 in EL4 lymphoma-bearing mice (Al-Ejeh et al., accompanying manuscript) together with our results using ^90^Y-DOTA-DAB4 in LL2 tumor-bearing mice indicate that this chemo-radioimmunotherapy is safest and most effective if given 24 hours after chemotherapy rather than immediately after chemotherapy. When ^111^In-DOTA-DAB4 was administered 24 hours after chemotherapy, both its tumor accumulation and blood clearance were accelerated. In comparision with its administration immediately after chemotherapy, we found that administering ^111^In-DOTA-DAB4 24 hours after chemotherapy resulted in significantly reduced accumulation of the radioligand in normal organs such as the gut (Al-Ejeh et al., accompanying manuscript), which is particularly susceptible to the cytotoxic effects of chemotherapy or ionizing radiation.

Although delaying administration of the radioligand accelerated its blood clearance and thus diminished its blood pool activity in normal organs, other factors may also account for the reduced normal organ accumulation of radioligand. For example, studies of gemcitabine-induced apoptosis of jejunal crypt cells indicate that apoptosis resolves by 24–36 hours post-treatment [39] perhaps because, in contrast to the apparently *inefficient* clearance of cells in malignant tissues, phagocytosis of apoptotic cells is *efficient* in normal tissues [40]. Consequently, we reason that the reduction in normal organ accumulation of radioactivity observed by delaying the injection of ^111^In-DOTA-DAB4 until 24 hours after cytotoxic chemotherapy will minimize the exposure of chemosensitive normal tissues such as gut and bone marrow to bystander toxicity emanating from any ^90^Y-DOTA-DAB4 bound to apoptotic cells in these tissues.

Confirmation of the therapeutic relevance of delaying the injection of the radioligand until 24 hours after chemotherapy was obtained when ^90^Y-DOTA-DAB4 administered to LL2 tumor-bearing mice 24 hours after chemotherapy produced superior antitumor efficacy compared with its administration immediately after chemotherapy. Therefore, we hypothesize that scheduling radioimmunotherapy 24 hours after chemotherapy improves the therapeutic ratio, and hence the clinical utility, of La-targeted chemo-radioimmunotherapy because the therapeutic ratio of any treatment will be improved by reducing its toxicity and/or increasing its efficacy at a given dose.

In all, we propose that several properties in combination contribute to the efficiency of La-directed radioimmunotherapy and make it superior to other previously described mAb therapies targeting dead cancer cells such as TNT, the antigen target of which is not induced by DNA-damaging chemotherapy in vitro. First, La is a universal and high-density target antigen that is overexpressed in malignant cells. It is also conditionally expressed in malignant cells by DNA-damaging therapy after dead normal cells have been cleared, and it appears to be durably retained in tumors. Second, arming DAB4 mAb with Yttrium-90 generates bystander killing, and then a self-amplifying recruitment of DAB4 that prolongs tumor retention of DAB4.

Furthermore, the ability of DNA-damaging treatment to create high tumor densities of La target antigen together with the prolonged intratumoral retention of DAB4 may extend the utility of this technology platform to other therapeutic modalities. For example, antibody-directed enzyme prodrug therapy [41], some antibody-drug conjugates [42], [43], functional nanoparticles [44], or genetically engineered T-lymphocytes [45], which like radionuclides may amplify therapeutic activity at the target binding site, can also exert bystander killing of tumor cells lacking the target antigen.

In conclusion, we provide preclinical proof-of-concept data for a unique form of systemic chemo-radioimmunotherapy that targets a universal tumor antigen and subsequently engenders a self-amplifying method of target creation to elicit a genotoxic chain reaction. Thus, this technology may meet the challenge of delivering higher tumor-directed doses of radioimmunotherapy particularly if used with radiosensitizing agents and/or new classes of pro-apoptotic agents [46]–[48]. If applied to treatment of distant metastases, this approach may deliver systemically the potentially curative benefits that radiosensitizing chemotherapy and external beam radiotherapy afford in treatment of locally advanced carcinomas of the head and neck, esophagus, lung, cervix, and rectum [49].

## Supporting Information

Figure S1 Effects of ^90^Y-DOTA-DAB4 alone or 24 h after cyclophosphamide and etoposide chemotherapy on body weight of EL4 tumor bearing mice. Data shown are percentage change in mean (±SEM) mouse weights (n = 5/group).
(0.03 MB DOC)

Figure S2 Biodistribution of ^90^Y-DOTA-DAB4 and ^111^In-DOTA-DAB4 in EL4 Tumor-Bearing Mice. B6 mice bearing syngeneic EL4 tumors were untreated or treated with full-dose chemotherapy, and given intravenous injections of ^111^In-DOTA-DAB4 (clear columns) or ^90^Y-DOTA-DAB4 (light gray columns) alone or ^111^In-DOTA-DAB4 (dark gray columns) or ^90^Y-DOTA-DAB4 (black columns) 24 h after chemotherapy. Mice were killed at 2, 24, 48, and 72 h after injection of radioimmunoconjugate to measure its accumulation in blood, main normal organs and tumors, which was calculated as the percentage of mass-normalized counts per minute (cpm) to total cpm of the injected dose at time 0 (%ID/g). Data shown are mean %ID/g±SEM (n = 5/group), *** P<0.001.
(0.06 MB DOC)

Table S1 Comparison of Tumor Doubling Time (TDT) and Combination Index (CI) in A, LL2, B, LNCaP, and C, Panc-1 tumor models. Tumor growth data from control and treated mice were fitted to exponential growth curves using GraphPad Prism (v.4.0) to generate tumor doubling times. Data are shown as mean±SEM. Combination index was calculated as (TDT for Chemo+RIT−TDT for control)/[(TDT for chemo−TDT for control)+(TDT for RIT−TDT for control)] e.g. for Chemo interaction with 0.46 MBq ^90^Y-DOTA-DAB4, CI = (h-a)/[(g-a)+(b-a)]. Note that CI>1 indicates supra-additive effects. A, As described in Figure 4 legend, LL2 tumor-bearing mice were (a) untreated (Control), treated with (b–f) ^90^Y-DOTA-DAB4 alone, (g) Chemo alone, or (h–l) ^1^24 h after Chemo, or (m) ^2^immediately after Chemo. B, As described in Figure 5 legend, LNCaP tumor-bearing mice were (a) untreated (Control), treated with (b) ^90^Y-DOTA-7E11 alone, (c) ^90^Y-DOTA-DAB4 alone, (d) Chemo alone, or (e) ^90^Y-DOTA-7E11 24 h after Chemo, or (f) ^90^Y-DOTA-DAB4 24 h after Chemo. C, As described in Figure 5 legend, Panc-1 tumor-bearing mice were (a) untreated (Control), treated with (b) ^90^Y-DOTA-DAB4 alone, (c) Chemo alone, or (d) ^90^Y-DOTA-DAB4 24 h after Chemo.
(0.05 MB DOC)
