# Supplementary material for: Chemotherapy Synergizes with Radioimmunotherapy Targeting La Autoantigen in Tumors
Source: PLoS One. 2009 Feb 27;4(2):e4630. doi: 10.1371/journal.pone.0004630 (PMC2645682; doi:10.1371/journal.pone.0004630)
Supplement: Table S1 — Comparison of Tumor Doubling Time (TDT) and Combination Index (CI) in A, LL2, B, LNCaP, and C, Panc-1 tumor models. Tumor growth data from control and treated mice were fitted to exponential growth curves using GraphPad Prism (v.4.0) to generate tumor doubling times. Data are shown as mean±SEM. Combination index was calculated as (TDT for Chemo+RIT−TDT for control)/[(TDT for chemo−TDT for control)+(TDT for RIT−TDT for control)] e.g. for Chemo interaction with 0.46 MBq 90Y-DOTA-DAB4, CI = (h-a)/[(g-a)+(b-a)]. Note that CI>1 indicates supra-additive effects. A, As described in Figure 4 legend, LL2 tumor-bearing mice were (a) untreated (Control), treated with (b–f) 90Y-DOTA-DAB4 alone, (g) Chemo alone, or (h–l) 124 h after Chemo, or (m) 2immediately after Chemo. B, As described in Figure 5 legend, LNCaP tumor-bearing mice were (a) untreated (Control), treated with (b) 90Y-DOTA-7E11 alone, (c) 90Y-DOTA-DAB4 alone, (d) Chemo alone, or (e) 90Y-DOTA-7E11 24 h after Chemo, or (f) 90Y-DOTA-DAB4 24 h after Chemo. C, As described in Figure 5 legend, Panc-1 tumor-bearing mice were (a) untreated (Control), treated with (b) 90Y-DOTA-DAB4 alone, (c) Chemo alone, or (d) 90Y-DOTA-DAB4 24 h after Chemo. (0.05 MB DOC) [file pone.0004630.s003.doc]

| **A.** | **Treatment** | **TDT (Days ± SEM)** | **CI** |
| --- | --- | --- | --- |
|  | 1. Control | 1.34 ± 0.02 |  |
|  | 1. 0.46 MBq | 1.34 ± 0.02 |  |
|  | 1. 0.92 MBq | 1.47 ± 0.04 |  |
|  | 1. 1.80 MBq | 1.51 ± 0.03 |  |
|  | 1. 3.60 MBq | 1.87 ± 0.02 |  |
|  | 1. 5.00 MBq | 2.14 ± 0.01 |  |
|  | 1. Chemo | 2.36 ± 0.02 |  |
|  | 1. Chemo + 0.46 MBq^1^ | 2.37 ± 0.02 | 1.01 |
|  | 1. Chemo + 0.92 MBq**^1^** | 3.15 ± 0.02 | 1.57 |
|  | 1. Chemo + 1.80 MBq**^1^** | 3.33 ± 0.02 | 1.67 |
|  | 1. Chemo + 3.60 MBq**^1^** | 3.91 ± 0.02 | 1.66 |
|  | 1. Chemo + 5.00 MBq**^1^** | 4.34 ± 0.01 | 1.72 |
|  | 1. Chemo + 5.00 MBq^2^ | 3.48 ± 0.01 | 1.11 |

| **B.** | **Treatment** | **TDT (Days ± SEM)** | **CI** |
| --- | --- | --- | --- |
|  | 1. Control | 1.81 ± 0.01 |  |
|  | 1. 7E11 | 1.99 ± 0.04 |  |
|  | 1. DAB4 | 2.92 ± 0.02 |  |
|  | 1. Chemo | 3.52 ± 0.01 |  |
|  | 1. Chemo+7E11 | 4.70 ± 0.02 | 1.52 |
|  | 1. Chemo+DAB4 | 13.72 ± 0.02 | 4.25 |

| **C.** | **Treatment** | **TDT (Days ± SEM)** | **CI** |
| --- | --- | --- | --- |
|  | 1. Control | 4.44 ± 0.02 |  |
|  | 1. DAB4 | 5.87 ± 0.04 |  |
|  | 1. Chemo | 4.88 ± 0.01 |  |
|  | 1. Chemo+DAB4 | 8.53 ± 0.02 | 2.19 |
