# Supplementary material for: Chemotherapy Synergizes with Radioimmunotherapy Targeting La Autoantigen in Tumors
Source: PLoS One. 2009 Feb 27;4(2):e4630. doi: 10.1371/journal.pone.0004630 (PMC2645682; doi:10.1371/journal.pone.0004630)
Supplement: Supplementary file 2 [file pone.0004630.s001.doc]

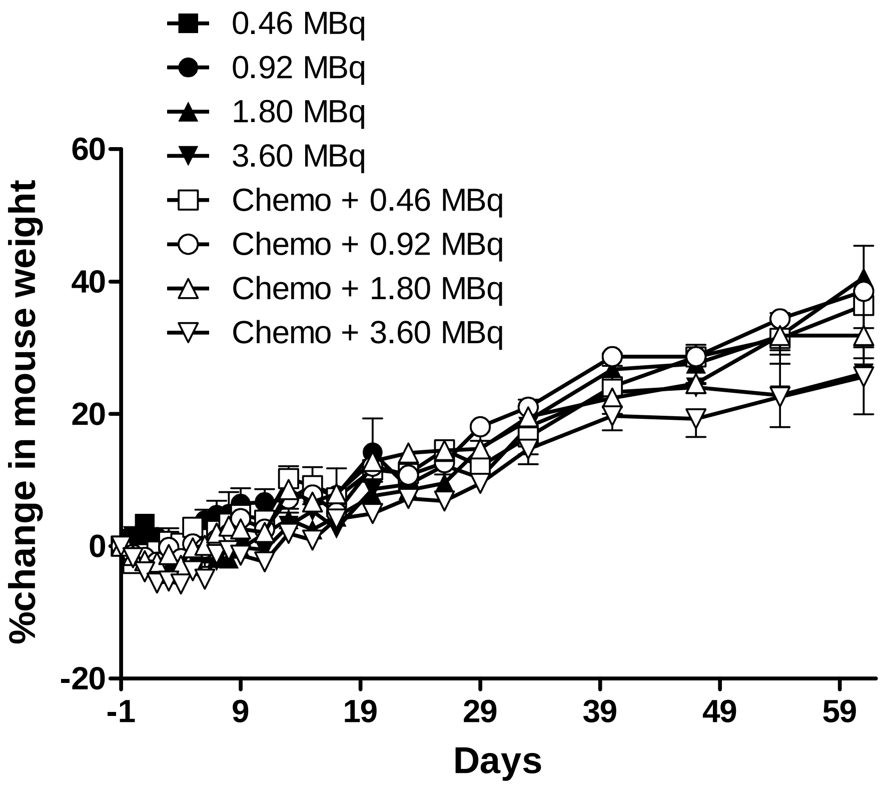

0.46 MBq
0.92 MBq
1.80 MBq
3.60 MBq
Chemo + 0.46 MBq
Chemo + 0.92 MBq
Chemo + 1.80 MBq
Chemo + 3.60 MBq
%change in mouse weight
60
40
20
0
-20
-1
9
19
29
39
49
59
Days
