# Supplementary material for: Chemotherapy Synergizes with Radioimmunotherapy Targeting La Autoantigen in Tumors
Source: PLoS One. 2009 Feb 27;4(2):e4630. doi: 10.1371/journal.pone.0004630 (PMC2645682; doi:10.1371/journal.pone.0004630)
Supplement: Supplementary file 3 [file pone.0004630.s002.doc]

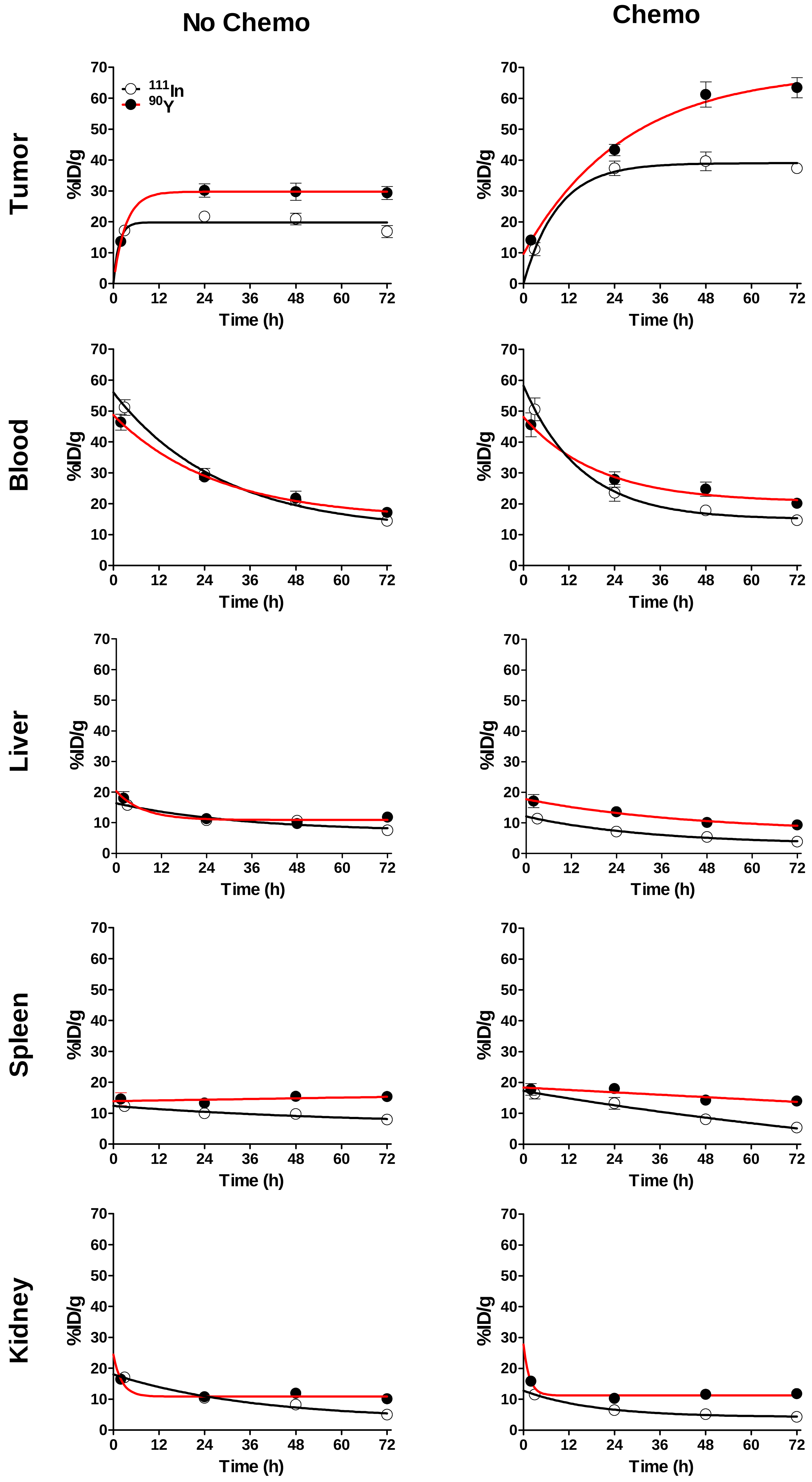
No Chemo
Chemo
Tumor
Blood
Liver
Spleen
Kidney
%ID/g
Time (h)
111In
90Y
0
12
24
36
48
60
72
10
20
30
40
50
60
70
